# Minimally invasive cytoreductive radical prostatectomy, exploring the safety and feasibility of a single-port or multi-port robotic platform

**DOI:** 10.1186/s12894-024-01463-2

**Published:** 2024-03-26

**Authors:** Daniel J. Lama, Kyle Thomas, Simone L. Vernez, Oluwatimilehin Okunowo, Clayton S. Lau, Bertram E. Yuh

**Affiliations:** 1 Division of Urology and Urologic Oncology, Department of Surgery, City of Hope Comprehensive Cancer Center, Duarte, CA USA; 2 Department of Computational and Quantitative Medicine, Division of Biostatistics, Beckman Research Institute of City of Hope, Duarte, CA USA

**Keywords:** Cytoreduction, Prostate cancer, Prostatectomy, Prostate-specific antigen, Robotic surgical procedures

## Abstract

**Background:**

Consolidative resection or cytoreductive radical prostatectomy (CRP) may benefit men with non-organ confined prostate cancer. We report the safety, feasibility, and outcomes of robot-assisted laparoscopic CRP using a single-port (SP) or multi-port (MP) platform.

**Methods:**

We reviewed consecutive men with clinical node positive or metastatic castrate-sensitive prostate cancer who underwent IRB-approved CRP and extended pelvic lymph node dissection using the da Vinci SP or MP Surgical Systems (Intuitive Surgical, Sunnyvale, CA) from 2015–2022. Perioperative data and Clavien-Dindo 90-day complications were recorded.

**Results:**

Twenty-four men with a median age of 61 (IQR 56—69) years and prostate-specific antigen of 32.1 (IQR 21.9—62.3) ng/mL were included. Clinical N1, M1, or N1 + M1 disease were detected in 8 (33%), 9 (38%), 7 (29%) patients, respectively. There was no difference in positive margins, 41% vs. 29% (*P* = 0.67), lymph node yield, 21 (IQR 14–28) vs. 20 (IQR 13.5–21) nodes (*P* = 0.31), or estimated blood loss, 150 mL (IQR 100–200) vs. 50 mL (IQR 50–125) (*P* = 0.06), between the MP and SP cohorts, respectively. Hospital length of stay was significantly shorter for the SP group, same-day discharge (IQR 0–0), compared to MP, 1-day (IQR 1–1), *P* < 0.001. One grade III bowel obstruction and lymphocele occurred in the MP cohort. No major complications occurred in the SP cohort.

**Conclusion:**

Robot-assisted laparoscopic CRP is safe and feasible for select men with advanced castrate-sensitive prostate cancer.

## Background

The concept of cytoreduction for palliation or treatment of metastatic cancer is practiced for several visceral malignancies including ovarian, gastrointestinal, and renal cell carcinoma [1–3]. The role of cytoreduction in metastatic prostate cancer (mPC) is largely experimental in humans, but murine models suggest reduced metastatic disease progression, reduced prostate specific antigen (PSA) velocity, and perhaps prolonged survival [4, 5]. Moreover, a national cancer registry investigation identified survival benefits for men undergoing cytoreductive radical prostatectomy (CRP) or whole gland radiation therapy in the metastatic setting [6]. In addition, there is evidence that CRP may offer symptomatic improvement and reduce the incidence of future complications from local progression such as bladder outlet obstruction [7, 8]. Similarly, in the setting of locally advanced prostate cancer (≥ cT3N+), there is retrospective evidence suggesting that radical prostatectomy or radiotherapy may improve outcomes such as recurrence and disease-specific survival [9]. It remains however, that concurrent radiation and hormonal therapy are the only interventions with level I evidence demonstrating a survival benefit in these settings [10].

The feasibility and oncologic outcomes of open CRP have been reported previously [11]; however, there is a paucity of data specifically studying a minimally invasive robot-assisted approach. At our tertiary referral center, CRP is offered in an experimental setting to men with clinical evidence of stage IV prostate cancer using either a multi-port (MP) or single port (SP) robotic platform. SP technology is relatively new, with Food and Drug Administration approval in 2018 for use in a variety of oncologic settings in urology [12]. Our aim was to summarize the safety, feasibility, and perioperative outcomes of CRP achieved using an SP or MP technology.

## Methods

### Patient selection

We reviewed an institutional review board (IRB) approved (#00149) prospective database of consecutive men who underwent robot-assisted radical prostatectomy (RARP) at our institution between 2015–2022. We then identified patients who had clinical evidence of stage IV prostate cancer and consented to cytoreductive surgery in the context of a clinical trial (IRB #14201, #14303, or #17347). We therefore define CRP as resectable primary tumor and American Joint Committee on Cancer clinical stage N1 and/or M1a-c disease following contrast or radionuclide enhanced cross-sectional imaging and/or tissue biopsy of a metastatic lesion. CRP was performed using the da Vinci MP or SP surgical system (Intuitive Surgical, Sunnyvale, CA) by experienced robotic surgeons with fellowship training. MP CRP was performed by four surgeons and the remaining seven SP CRPs were performed exclusively and non-selectively by a single surgeon with more than 50 prior SP RARPs for organ-confined disease. All patients were evaluated in our institutional multidisciplinary clinic prior to CRP. Shared decision-making was performed prior to obtaining informed consent for IRB enrollment, with the objective of determining the non-curative clinical benefits of CRP. Patients received ADT monotherapy, ADT + abiraterone/prednisone, or proceeded directly to CRP. Men with poor performance status, i.e., Eastern Cooperative Oncology Group score ≥ 2, and estimated life expectancy < 5-years were excluded.

### Operative technique and functional outcomes

Transperitoneal CRP via a posterior approach and extended pelvic lymph node dissection (ePLND) were performed in similar fashion irrespective of robotic platform. No assistant ports were used for SP CRP. Wide resections of the prostate at the bladder neck and lateral margins were utilized, and no nerve-sparing was performed. In the event of oligometastatic disease, concurrent or delayed metastastectomy was not performed. All patients were transferred to the recovery unit for several hours and reassessed to determine appropriateness for same-day discharge. Minor (≤ grade II) and major (≥ grade III) 90-day complications were recorded using a modified Clavien-Dindo classification system [13]. Functional outcomes included continence, which was defined as no pad use or use of a security liner. Sexual function assessment was not performed, as nearly all men were impotent due to the presence of adjuvant ADT.

### Statistical analysis

Primary endpoints included perioperative outcomes (operative time, blood loss, lymph node yield, positive margin rate, and length of hospital stay) and Clavien-Dindo complications. Secondary endpoints included continence. Basic demographic, clinical, and surgical characteristics were collected. The study cohorts were described using summary statistics: median, interquartile range (IQR) and counts (%). We calculated the age-adjusted Charlson Comorbidity Index up to the date of CRP excluding history of prostate cancer or mPC from the calculation since they are the primary conditions of interest. Of note, none of the patients had a different malignancy [14]. Differences in characteristics between MP and SP operative groups were assessed with the Chi square (χ2) or Fisher’s exact test for categorical variables, and Wilcoxon rank-sum test for continuous variables. A *P* value < 0.05 was considered statistically significant. Data management and statistical analyses were conducted using SAS version 9.4 (SAS Institute Inc., Cary, NC, USA) and R version 4.2.1 (R Foundation for Statistical Computing).

## Results

We identified 24 men with a median PSA of 32 (IQR 21.9–62.3) ng/mL who underwent CRP, 17 (71%) men using the MP and 7 (29%) men using the SP platform. Median follow-up was 2.6 (IQR 0.6–4.3) years. Baseline characteristics were similar between groups (Table 1). ADT was given to 7 (41%) MP patients (one patient received ADT + docetaxel) and 6 (86%) SP patients. Clinical N1, M1, or N1M1 was detected in 8 (47%) and 4 (57%), 4 (24%) and 0 (0%), and 5 (29%) and 3 (43%) of patients in the MP and SP cohorts, respectively. Cumulative median biopsy Gleason score was 8 (IQR 7–9) for patients undergoing CRP.

**Table 1:** Preoperative characteristics

		**Surgery type**		
**Characteristic**	**Overall** **(*n* = 24)**	**Single-port** **(*n* = 7)**	**Multi-port** **(*n* = 17)**	***P-*value**
**Age, median (IQR), year**	61 (56–69)	55.5 (51.3–63.5)	61 (57–70)	0.23
**Race, No. (%)**				> 0.99
Asian	4 (17)	1 (14)	3 (18)	
African American	4 (17)	1 (14)	3 (18)	
Hispanic	4 (17)	2 (29)	2 (12)	
Caucasian	12 (50)	3 (43)	9 (53)	
**ASA, No. (%)**				0.53
**II**	11 (46)	2 (29)	9 (53)	
**III**	11 (46)	4 (57)	7(41)	
**IV**	2 (8)	1 (14)	1 (6)	
**Age-adjusted CCI, median (IQR)**^**a**^	2 (1–3)	2 (1–3)	2 (2–3)	0.79
**Operative time, median (IQR), min**	247 (214.5–287)	281 (223.5–290)	237 (210–283)	0.49
**Biopsy Gleason score, median (IQR)**	8 (7–9)	8 (7–8.5)	8 (7–9)	0.35
**Prostate vol., median (IQR), cc**^**b**^	47 (35.3–58.4)	38.5 (32–53.5)	49 (36.5–58)	0.41
**Clinical T stage, No. (%)**				0.22
T1c	3 (13)	1 (14)	2 (12)	
T2a	2 (8)	0 (0)	2 (12)	
T2b	3 (13)	1 (14)	2 (12)	
T2c	5 (21)	0 (0)	5 (29)	
T3a	6 (25)	4 (57)	2 (12)	
T3b	5 (21)	1 (14)	4 (24)	
**Clinical N stage, No. (%)**				0.28
N1	20 (83)	7 (100)	13 (76)	
N0	4 (17)	0 (0)	4 (24)	
**Clinical M stage, No. (%)**				> 0.99
M1	12 (50)	3 (43)	9 (53)	
M0	12 (50)	4 (57)	8 (47)	

*ASA* American Society of Anesthesiologists, *CCI* Charlson Comorbidity Index, *IQR* interquartile range

^a^Patient age at surgery was assigned a weight, where a score of 1 was added for every decade over 40-years. The scores from each comorbidity of CCI along with the weighted scores for age were added to calculate the age-adjusted CCI

^b^Prostate volume derived from whole gland weight at the time of cytoreductive radical prostatectomy

Median procedure time was 247 (IQR 214.5–287) minutes and similar between groups (*P* = 0.49). There were no conversions to open surgery. Although estimated blood loss was lower in the SP cohort, 50 mL (IQR 50–125), compared to the MP cohort, 150 mL (IQR 100–200), the difference did not reach statistical significance (*P* = 0.06). Importantly, median ePLND yield was similar between groups, 21 (IQR 14–28) versus 20 (IQR 13.5–21) lymph nodes for MP and SP, respectively.

Eight (53%) patients were found to have Gleason grade group 4 or 5 disease, 16 (67%) were found to have ≥ pT3 disease, and nine (38%) patients had positive margins. Positive margin rate was 41% in the MP group and 29% in the SP group. Eighteen (75%) were confirmed pN1. Two patients (12%) in the MP group who had neoadjuvant ADT achieved a complete pathologic response. There were no differences between MP and SP with respect to pathologic outcomes (Table 2). Ten MP patients (59%) and two SP patients (29%) required salvage or adjuvant radiation therapy for positive margins/adverse pathology or rising PSA. A summary of adjuvant therapies in both groups in shown in Table 3.

**Table 2:** Perioperative characteristics

		**Surgery type**		
**Characteristics**	**Overall** **(*n* = 24)**	**Single-port** **(*n* = 7)**	**Multi-port** **(*n* = 17)**	***P-*value**
**Pathology grade, No. (%)**				0.22
3 + 4	3 (13)	2 (29)	1 (6)	
4 + 3	4 (17)	1 (14)	3 (18)	
4 + 4	3 (13)	0 (0)	3 (18)	
4 + 5	4 (17)	0 (0)	4 (24)	
5 + 4	1 (4)	0 (0)	1 (6)	
Indeterminate^a^	9 (37)	4 (57)	5 (29)	
**pT stage, No. (%)**				0.18
pT0	2 (8)	0 (0)	2 (12)	
pT2	4 (17)	2 (28)	2 (12)	
pT2c	2 (8)	0 (0)	2 (12)	
pT3a	4 (17)	3 (43)	1 (6)	
pT3b	11 (46)	2 (29)	9 (53)	
pT4	1 (4)	0 (0)	1 (6)	
**pN stage, No.(%)**				0.36
pN0	6 (25)	3 (43)	3 (18)	
pN1	18 (75)	4 (57)	14 (82)	
**EBL, median (IQR), mL**	100 (50–200)	50.0 (50–125)	150 (100–200)	0.064
**Total lymph nodes, median (IQR)**	20.5 (14–25)	20.0 (13.5–21)	21 (14–28)	0.31
**Positive lymph nodes, median (IQR)**	1.5 (0.8–6.8)	1 (0–2.5)	3 (1–9)	0.11
**Surgical margins, No. (%)**				0.67
Negative	15 (63)	5 (71)	10 (59)	
Positive	9 (38)	2 (29)	7 (41)	

*EBL* estimated blood loss, *IQR* interquartile range

^a^Histopathologic Gleason score could not be assigned postoperatively due to treatment effect of androgen deprivation therapy

**Table 3:** Adjuvant therapies in the multi-port and single-port cohorts following cytoreductive radical prostatectomy

		**Surgery type**	
Adjuvant therapy	**Overall (*n* = 24)**	**Single-port (*n* = 7)**	**Multi-port** **(*n* = 17)**
**ADT, No. (%)**	19 (79)	6 (86)	13 (77)
**Systemic chemotherapy, No. (%)**	1 (4)	0 (0)	1 (6)
**Radiation therapy, No. (%)**	1 (4)	2 (29)	8 (47)
**CYP17A inhibitor, No. (%)**	2 (8)	2 (29)	6 (35)
**Androgen signaling inhibitor, No. (%)**^**a**^	1 (4)	0 (0)	5 (29)

*ADT* androgen deprivation therapy

^a^Androgen signaling inhibitors include bicalutamide, darolutamide, and enzalutamide

All SP patients were evaluated postoperatively and determined appropriate for same day discharge. Accordingly, the median length of stay was significantly shorter in the SP compared to MP cohort, median 0 (IQR 0–0) vs. 1 (IQR 1–1) day (*P* < 0.001), respectively. Ten complications were seen in total with no difference in the rate of complications between groups (Table 4). The overall major complication rate was 8%, i.e., one MP patient had small bowel obstruction requiring surgical decompression (grade III) and another had a lymphocele requiring percutaneous intervention (grade III). Four (24%) patients in the MP cohort died of mPC a median 3.3 (IQR 2.9–3.9) years after CRP. Of the patients who were continent before surgery, 10/13 (77%) MP and 2/6 (33%) SP patients remained continent after surgery (*P* = 0.13).

**Table 4:** 90-day Clavien-Dindo complications

		**Surgery type**		
Characteristics	**Overall (*n* = 23)**^**a**^	**Single-port (*n* = 7)**	**Multi-port (*n* = 17)**	***P*-value**
**Anastomotic leak, No. (%)**	2 (9)	0 (0)	2 (12)	> 0.99
**Lymphocele, No. (%)**	1 (4)	0 (0)	1 (6)	> 0.99
**Intestinal obstruction, No. (%)**	1 (4)	0 (0)	1 (6)	> 0.99
**Ileus, No. (%)**	2 (9)	0 (0)	2 (12)	> 0.99
**Neuropathy, No. (%)**	1 (4)	0 (0)	1 (6)	> 0.99
**Bladder spasm, No. (%)**	1 (4)	1 (17)	0 (0)	0.26
**Atelectasis, No. (%)**	1 (4)	0 (0)	1 (6)	> 0.99
**Postop pain, No. (%)**	1 (4)	0 (0)	1 (6)	> 0.99

^a^One patient had insufficient follow-up to determine 90-day complications

## Discussion

The stage migration of men with newly diagnosed prostate cancer has resulted in an increased incidence of patients presenting with de novo metastatic disease and an unfavorable impact on disease specific mortality [15, 16]. For these patients, long-term systemic hormonal therapy is the foundation of treatment. The historic role of CRP has challenged this dogma, advocating that select men may incur a survival or symptomatic benefit from cytoreduction. At our institution, we agree that such benefits do exist and hypothesize that minimally invasive surgery is key to mitigate the morbidity and mortality of such an endeavor. In an effort to substantiate our hypothesis and to study the evolution of robotic surgical technology we performed the first feasibility study of CRP using either an SP or MP robotic platform.

The role of cytoreduction in mPC remains exploratory pending the outcomes of prospective trials (NCT01751438, NCT03655886). The biologic basis of cytoreduction is theorized to be the reduction in the circulating tumor cell (CTC) burden, cytokine signaling, and other associated oncologic promoting factors in men with metastatic cancer. A recent systematic review of clinical applications of CTC detection found prognostic significance of CTC concentration with respect to progression-free survival, overall survival or response to androgen inhibition specifically in mPC [17]. For many patients that progress to castrate resistance, CTCs have been shown in a post hoc analysis to be an independent predictor of overall survival following treatment [18]. Indeed, Mandel et al. studied the pre- and postoperative significance of CTCs for 33 men undergoing CRP for hormone-naïve oligometastatic PC with respect to castration resistant-free and overall survival [19]. After a median follow-up period of ~ 3-years the authors found CTC thresholds predicting shorter time to castrate resistance and impacting overall survival after CRP. Furthermore, they suggest CTCs can be a reliable index for CRP patient selection and initiation of subsequent secondary therapies postoperatively. Thus, palliative effects of CRP cannot be measured in isolation and likely require additional clinical variables to optimize subsequent management.

Although CRP remains largely investigational, prior studies demonstrate symptomatic and oncologic benefits despite the natural history of mPC. Heidenreich et al. compared the effects of open CRP in a carefully selected cohort of 23 patients who were given neoadjuvant ADT and had evidence of low-volume non-visceral metastases to a matched group of 38 controls treated with continuous ADT [7]. The authors report significantly improved progression-free (38.6 vs. 26.5-months) and cancer-specific (95.6% vs. 84.2%) survival, as well as latency to castrate-resistance (40 vs. 29-months) for men treated with CRP or ADT, respectively. In addition, 29% of controls required palliative interventions for symptoms related to lower or upper urinary tract progression that was avoided by radical whole gland treatment. CRP was not without adjuvant therapies, as ~ 56% of patients with positive margins required radiation to the prostate bed and 30% required additional hormonal and/or chemotherapy after median follow-up of 35-months. Poelaert et al. performed a multi-center prospective trial to define outcomes of CRP as primary treatment versus standard of care ADT [20]. Despite CRP, 76% (13/17) of patients required delayed ADT ± chemotherapy. However, after approximately 36-months 49% (13/29) patients managed without CRP progressed to castrate-resistance and 24% (7/29) died from mPC. Moreover, retrospective studies conducted through the Surveillance Epidemiology and End Results (SEER) database compared the effects of CRP or radiation therapy to patients without either whole gland intervention and reported similar palliative effects on cancer progression. Culp et al. found that CRP resulted in improved disease-specific (76%) and 5-year overall (67%) survival, outcomes that remained significant regardless of metastatic burden (M1a-c) when compared to patients likely managed with ADT (23% and 49%, respectively) [21]. Satkunasivam et al. performed a similar analysis of SEER mPC patients treated with CRP or radiation therapy versus no local intervention, and after adjustment for several clinicopathologic variables, noted lower cancer-specific (HR 0.48) and all-cause (HR 0.43) mortality for patients treated with radical prostatectomy [6]. Collectively, these findings assert likely multifactorial benefits to CRP that merit further prospective randomized analysis.

We required relatively fit men to provide informed consent for CRP as eligibility criteria. Prior studies of CRP allude to careful patient selection to properly screen men for the procedure. Characteristics such as biopsy Gleason score ≤ 7, prior response to ADT (nadir PSA ≤ 1 ng/mL), absolute preoperative PSA ≤ 20 ng/mL, younger age (age ≤ 70), and low metastatic burden portend improved oncologic outcomes following CRP [6, 7, 20, 21]. To control for an inherent risk of selection bias Steuber et al. prospectively collected data for 43 men who underwent CRP versus 40 men managed with systemic hormonal therapy and required identical inclusion criteria, that is, asymptomatic low-volume osseous metastases with PSA < 150 ng/mL and no prior radiation [17]. The authors reported no difference in castrate-resistance or overall survival but cited a 7 vs. 35% difference in complications related to local progression. Therefore, future inclusion criteria of CRP must weigh oncologic benefit with symptomatic control.

We report similar oncologic and perioperative outcomes relative to prior studies of CRP. Our notable overall positive margin rate falls within range of that reported previously (14–82%) [7, 20] and speaks to the technical challenge of non-curative radical surgery. Alongside primarily ≤ grade II complications, we identified two (8%) patients who sustained major complications, albeit in a small total number of patients, and no grade IV-V complications occurred. These findings contrast the two grade II complications cited in Poelart et al.’s robot-assisted CRP study and a 42% ≥ grade III complication rate following open CRP reported by Hendrich et al. [7, 20]. However, outside of a clinical trial setting and from a risk/benefit standpoint, men with mPC are generally not given surgery as a treatment option. We sought to offer the least invasive means of performing cytoreduction to help patients recover faster without sacrificing safety or extent of surgery.

SP surgery has the potential benefits of reduced incisions, less pain, and faster recovery compared to MP surgery [22]. Criticisms of SP surgery include limited instrumentation and ability to retract, yet the single trocar system and double-articulating instruments facilitate maneuverability into a number of surgical approaches to RARP [23, 24]. This suggests that despite concerns over the technical challenges of the SP platform, SP may be as safe and versatile as MP CRP. Lastly, there are few, if any, studies reporting both the operative outcomes of SP and MP approaches in this setting.

The present study has several limitations. Foremost, is its small sample size and the lack of a formal power analysis to detect any statistically significant differences between robotic platforms. In addition, without randomization or propensity score matching our cohorts are subject to selection bias, although we found no baseline differences between groups including the incidence of patients with locally advanced or metastatic disease. Furthermore, without a non-surgical control group we cannot report the differences in complications, oncologic, or functional outcomes of a standard of care approach. In addition, our perioperative outcomes cannot be generalized in the setting of an exceedingly rare procedure, limited access to robotic technology, and the degree of surgeon experience required [12]. We reported a difference in postoperative recovery between robotic platforms, yet the experience gained from completing more than two-thirds of MP cases beforehand may have influenced the treatment team’s performance completing SP operations. Additionally, the criteria used to determine discharge readiness is subject to best clinical judgement and could not be cited for reproducibility. Therefore, external validation of robot-assisted CRP is necessary to make conclusions as to the risks and benefits of surgical intervention. Lastly, we report solely perioperative outcomes but lack the oncologic and survival data, e.g., PSA response, progression-free and overall survival, necessary to sufficiently scrutinize the role of MP or SP CRP for patients with metastatic castrate sensitive PC. These outcomes, in the setting of high positive margin (38%) and incontinence rates (33–77%) require further investigation to properly inform patients of the risks, benefits, and quality of life impact of CRP.

## Conclusions

Minimally invasive CRP is safe, feasible, and results in similar perioperative outcomes when using an MP or SP robotic surgical platform. Further analysis of oncologic outcomes of CRP is necessary to elucidate the potential benefits further use of robotic surgical technology for such an intervention.

## Data Availability

The datasets used and/or analyzed during the current study are available from the corresponding author on reasonable request.

## Abbreviations

ADT: Androgen deprivation therapy
CRP: Cytoreductive radical prostatectomy
ePLND: Extended pelvic lymph node dissection
mPC: Metastatic prostate cancer
MP: Multi-port
PSA: Prostate-specific antigen
SEER: Surveillance Epidemiology and End Results
SP: Single-port
